# Atropine Intoxication

**Published:** 1869-08-15

**Authors:** 


### *Atropine Intoxication.*

A curious case of delirium has recently occurred in the service of M. Richet (Paris), the subject being an old man, into whose eyes a half milligramme (about .0075 of a grain) of atropine in solution had been dropped twice daily for a week.

The delirium resembled the subdelirium of typhoid fever, the patient being able to reply to questions, when his attention was fixed. This condition was at first supposed to be due to purulent infection from a recent anthax, and sulphate of quinine was administered, especially as the delirium presented very marked exacerbations at the same hour of each day. Under this treatment the patient became so violent as to necessitate the application of the strait-waistcoat. Upon the discovery of the real cause, the use of the atropine was discontinued, and the morbid condition disappeared immediately.

The *Gazette des Hopitaux* urges the necessity of extreme caution in the administration of powerful therapeutic agents in the cases of unusually nervous people, the anæmic, children, and old men, in whom nervous equilibrium is less stable.

M. Richet also narrates in the same journal (June 26, 1869) a case of acute cystitis, asserted to be produced by the exhibition of *silica of the thirtieth dilution* by a homœopathist. When the discovery was made that the patient had a blistered surface which he dressed daily with cantharidine ointment, and this had been removed, the cystitis disappeared and the *silica* ceased to reproduce it.

Dr. E. Brown-Sequard communicated to the Imperial Academy of Medicine, Paris, at its meeting March 16th, 1869, two interesting facts observed by him in some recent experiments, in consequence of injuries to the restiform bodies. The first fact referred to is the occurrence of hæmorrhage under the skin of the ear; the second is dry gangrene of the ears.

A third fact observed by the distinguished experimenter is that lesions of the sciatic nerves induce absolutely the same results as do those of the spinal cord.

Ingestion of three grammes (three-fourths of a drachm) of croton oil by a child of six years. Violent vomiting, slight diarrhœa, rapid recovery.—Dr. Manvezin, *Gazette des Hopitaux.*

A little girl six years of age, affected with a slight impetigo of the upper lip, took, by mistake, and not without great objection, three grammes of oil of croton tiglium in the morning, fasting, in a cup of coffee. She complained of the detestable taste of the medicine and of the pricking which she felt at the isthmus of the fauces, even at the moment of swallowing. Soon afterwards she experienced acute pain in the epigastrium, speedily followed by violent and very profuse vomitings, during nearly three-quarters of an hour.

After having vomited she sank into a profound sleep remaining in that condition for four hours, at the end of which she asked for food. Her parents gave her some soup; the child felt no more pain either at the epigastrium nor in the abdomen. She had but two scanty, loose evacuations of the bowels.

The next day I saw the child; she did not appear indisposed; the throat was not red; no eruption was perceptible; but at the commissure of the lips, on the right upper eyelid, and upon the left buttock were some patches of minute vesicular eruption, such as croton oil ordinarily produces, and doubtless caused by contact of the hands of the child, smeared with the vomited matter, with those parts of the body.

The oil swallowed was proved to be of excellent quality, and produced upon the skin, in a very little time, an abundant vescicular eruption. How is this apparent harmlessness to be explained? Why were there not more numerous evacuations?

The violent vomiting doubtless expelled the greater portion of the oil; but did there not remain in the stomach *a single drop*, a dose usually sufficient to produce violent purgation in a child of that age? How far did the soup, taken *four hours after the ingestion of the oil*, contribute to neutralize the action of the poison? are questions worthy of consideration.

The medical societies of London are about to fuse in order to form an Academy of Medicine.

The initiative has been taken by the Medico-Chirurgical Society, and the Obstetrical Society is about to follow its example.—*Gaz. des Hop.*

A Pharmaceutical Congress, to which representatives from all *civilized nations* are invited, will take place in Vienna in September next.

Several important questions of public interest will be discussed. Amongst others the subject of a universal Pharmacopœia, with the view to obviate the difficulties in the preparation of foreign prescriptions with medicines prepared according to another pharmacopœia.

